# Shared clinical governance arrangements between NHS and independent acute hospitals in England: Findings from a national survey of senior leaders

**DOI:** 10.1177/13558196261431315

**Published:** 2026-03-19

**Authors:** Gemma Stringer, Jane Ferguson, Kieran Walshe, Christos Grigoroglou, Thomas Allen, Michael Anderson, Karen Bloor, Eleanor Gee, Nils Gutacker

**Affiliations:** 1 Division of Population Health, Health Services Research and Primary Care, The University of Manchester, Manchester, UK; 2 Alliance Manchester Business School, The University of Manchester, Manchester, UK; 3 Health Services Management Centre, University of Birmingham, Birmingham, UK; 4 Manchester Centre for Health Economics, Division of Population Health, Health Services Research and Primary Care, University of Manchester, Manchester, UK; 5 Danish Centre for Health Economics, University of Southern Denmark, Denmark; 6 Health Organisation, Policy, and Economics (HOPE), Division of Population Health, Health Services Research and Primary Care, University of Manchester, Manchester, UK; 7 Department of Health Sciences, 8748 University of York, York, UK; 8 Centre for Health Economics, 8748 University of York, York, UK

**Keywords:** clinical governance, quality and safety, national survey

## Abstract

**Objectives:**

To present the findings from a national survey of senior leaders in NHS and independent hospitals in England concerning the effectiveness of shared arrangements for clinical governance. To provide a comprehensive overview of shared arrangements for the oversight of consultants’ practice, processes for appraisal and revalidation, and the management of significant concerns. The results from this study will improve understanding of the practical functioning of clinical governance processes at the interface between the NHS and the independent sector.

**Methods:**

Between December 2023 and April 2024, an online survey was distributed to senior leads with governance responsibilities in NHS and independent hospitals in England.

**Results:**

320 responses were received (response rate 42%), 235 from individuals working in NHS trusts (response rate 40%) and 85 from individuals in independent hospitals (response rate 48%). Respondents reported that some clinical governance arrangements are established across both sectors, with some relationships characterised as positive and relatively strong. However, relationships often depended on goodwill, personal connections, and consultant probity, rather than the systematic implementation of recommended processes. Respondents expressed concerns regarding the non-mandatory and unregulated nature of processes for sharing concerns, believing this led to insufficient resources and challenges in verifying information. They called for improved data quality, better communication and information sharing and more robust and formalised processes.

**Conclusions:**

Shared clinical governance arrangements between the NHS and independent sectors are in place in some but not all of the organisations where respondents’ consultants worked. This raises concerns about progress towards implementing the Paterson inquiry recommendations, including access to consultants’ whole practice information and sharing concerns about consultants working across different providers. The findings may also hold relevance for international audiences where medical staff work across multiple healthcare providers. Further empirical research is needed to compare clinical governance arrangements between the NHS and independent sectors, and suggest how shared clinical governance can be organised to assure the quality and safety of care.

## Background

There are longstanding concerns among policymakers and leaders in the NHS and independent healthcare sectors about differences in the quality and safety of patient care between sectors, and the effectiveness and impact of shared arrangements for clinical governance. A number of reports and inquiries, such as the Paterson inquiry,^1,2^ the medicines and medical devices safety review,^3^ a recent Healthcare Safety Investigation Board report^4^ and the Care Quality Commission state of care report,^5^ have highlighted the need for strong, shared arrangements to improve clinical governance and ensure high quality patient care. These issues extend beyond the healthcare system in United Kingdom; they are relevant to other countries where healthcare professionals frequently operate across multiple providers and there is a need for shared information about clinical governance to ensure the quality and safety of care.^6,7^

A growing volume of NHS funded care, particularly in elective surgery, is delivered in independent hospitals, and pathways of care increasingly cross both sectors.^8^ This is largely delivered by a shared clinical workforce, with most consultants working in independent hospitals having a substantive appointment in the NHS. The proportion of elective care delivered by independent sector providers (ISPs) in England varies, but as of 2022, the independent sector provided around 9% of all NHS-funded elective procedures.^7,8^ For hip and knee replacements and cataract surgeries, around one in three NHS-funded procedures are now performed by the independent sector.^9^ In 2020/21, the NHS allocated £11.8 billion for services rendered by ISPs, an increase of £2 billion on the previous year.^10^ Successive administrations in England have increasingly adopted a mixed economy of care, aimed at fostering competition, enhancing patient choice, expanding capacity for elective care, and improving efficiency.^11^ The degree to which these policy goals have been achieved is contested.^12,13^

Clinical governance refers both to the systems that organisations put in place to assure and improve the quality of clinical care, and the accountability of organisations for how those systems function.^14^ It includes processes to report concerns to regulators, identify poor practice, share information between NHS and independent sectors, and manage patient complaints. Clinical governance arrangements are designed to keep patients safe within and across NHS and independent hospitals. Broader models of healthcare governance encompass multiple interrelated levels of oversight and accountability. At the organisational level, board governance provides strategic leadership and assurance that systems of clinical governance are implemented, resourced and aligned with statutory obligations and quality and safety goals.^15,16^ At the professional level, self-regulation, via mechanisms such as appraisal, revalidation and adherence to professional standards, supports clinical competence and ethical practice.^17,18^ Effective shared clinical governance between NHS and independent hospitals therefore depends not only on operational processes for information exchange, but also on how these wider governance structures interact.

Serious issues with consultants harming patients in their work across the NHS and independent sector are rare but some high-profile cases^2,19–21^ have emphasised the need for robust clinical governance arrangements between the sectors, with established communication channels so that information can be shared quickly and effectively. Work has been done to improve the robustness of clinical governance arrangements across the NHS and independent healthcare sectors. The Care Quality Commission, which regulates both NHS and independent hospitals, published a report based on its inspections of 206 independent hospitals in 2018.^5^ Quality and safety of care was generally high but monitoring of clinical governance was not consistently robust. In response, the Independent Healthcare Providers Network (IHPN) convened an expert group to develop the Medical Practitioners Assurance Framework (MPAF) published in 2019 and refreshed in 2022.^22,23^ It provides a framework for clinical governance in independent hospitals to improve the quality of care and safety for patients. Alongside recommendations to improve clinical governance within the independent sector, the MPAF also emphasised the importance of improvements in collaboration between the sectors, for example communicating any concerns about medical practitioners to all organisations where they practice, and addressing confusion about what and how information can be shared about medical practitioners to support whole practice appraisal.^23^

Most medical consultants who work in independent hospitals work primarily in an NHS hospital, which has legal responsibility for oversight of their whole scope of practice, including work in the independent sector.^24^ Dual medical practice, when doctors practice in both public and independent hospitals simultaneously, has long existed in healthcare systems around the world, including in the UK since creation of the NHS, but it remains scantly debated and researched.^25^ Dual practice has implications for medical revalidation and the reporting of whole scope of practice. Reforms to medical regulation introduced in 2012 require all doctors and their employing organisations to undergo a periodic review to ensure they are up to date and fit to practice (called medical revalidation).^26^ A key role in this process is the responsible officer (RO). The RO is a senior doctor who ensures that doctors meet national standards, and makes recommendations to the General Medical Council (GMC), the independent regulator of doctors, physician associates and anaesthesia associates in the UK, about the fitness to practise of doctors connected to them, usually once every 5 years.^24^ Research on the implementation of medical revalidation has highlighted the difficulties of reporting on the whole scope of practice for doctors who work across NHS and independent hospitals, and the need for shared clinical governance arrangements.^27^

These issues reflect broader research showing that effective clinical governance depends not only on formal accountability systems but also on professional norms and leadership.^28,29^ A number of reports have made recommendations concerning the way NHS and independent hospitals should work collaboratively to improve clinical governance^1–5^ but we do not know whether or how those recommendations have been implemented or how those governance processes work in practice and are experienced, particularly at the interface between the sectors. The overall aim of this survey was to provide evidence on the perceived effectiveness of shared arrangements for clinical governance between NHS and acute independent hospitals to better understand the quality and safety of patient care.

## Method

### Survey design

The initial survey items were developed based on a detailed literature review of established guidance and inquiries relevant to cross sector medical practice governance, including the Paterson inquiry and the refreshed Medical Practitioners Assurance Framework (MPAF).^1,2,23^ A draft of the survey was sent to stakeholders and followed up with an interview to explore how clinical governance operates across the NHS and independent sectors, including how consultants’ scope of practice and patient pathways are managed. We explored stakeholder understanding of the question intent, the terminology used and appropriateness of the questions.^30,31^ Stakeholders involved in the survey development from the NHS included: one medical director, three chief medical officers and one NHS consultant. Contributors from the independent sector included: two clinical directors, one chief nurse, and three clinical governance leads. Other stakeholders involved included, 14 members of our project advisory group and eight members of our patient and public involvement group. The process of revising the survey items included stakeholder and project team review, question reworking, re-testing, and final revisions.

We produced an online cross-sectional survey comprising 56 questions using Qualtrics software (Online Supplement). Information was gathered about clinical governance processes between NHS hospitals and independent hospitals across the following categories, determined inductively as part of the survey development process: whole scope of practice appraisal and revalidation, dealing with significant concerns, understanding capacity and capability, sharing patient information for elective care and transfers and the impact of the COVID-19 pandemic (Supplemental Material contains the survey and additional results).

The survey followed standard reporting guidelines^32^ (see Online Supplement for further information) and received research ethics approval from the National Research Ethics Service in England (23/EE/0104). Agreement to take part was implicit through completion.

### Survey distribution

An initial contact list of staff with an executive lead role for clinical governance (who we refer to as senior leaders throughout the paper), including: chief executives; chief medical officers; chief nurses; clinical governance leads; directors of clinical services; directors of nursing; hospital directors; matrons; medical directors and RO’s. We obtained contact information from the General Medical Council (GMC), the Care Quality Commission (CQC), NHS England and a commercial UK healthcare contact data provider Correspondence with contacts to inform them of the research and to encourage engagement allowed us to refine the contact list, amending incorrect details and removing people who indicated they were not responsible for clinical governance at their organisation, had changed roles or had left the organisation. The personalised survey link and reminders were distributed via email to validated contacts–769 people (593 senior leaders from NHS trusts and 176 senior leaders from independent hospitals in England). The electronic link to the survey was active for 4 months between 11th December 2023 and 30th April 2024.

### Survey analysis

The survey was analysed in Stata 14^33^ using frequency tables to describe numeric and Likert scale data. Mann Whitney U test was used to explore differences in ratings of clinical governance arrangements by sector. We used thematic analysis to analyse the written responses to 14 questions about clinical governance arrangements and any suggested improvements, including obtaining information about scope of practice, sharing information about concerns, restrictions and referrals, and sharing patient information and managing patient transfer.^34^ Patterns of shared meaning were identified utilising an inductive approach where coding and theme development is based on the information found in the responses. Respondents’ free-text comments were brief and were used to supplement the findings from associated quantitative questions.^35^ Potentially identifying details in free-text comments were removed. Familiarisation with the free-text data was achieved through iterative reading and for each question possible codes were noted, taking account of repeated words or meaning. All written comments were reviewed holistically and overarching themes were mapped, encompassing the key messages across the data.

## Results

### Responses and characteristics

Of the 593 NHS leads and 176 independent sector leads, 320 usable responses were received (response rate 42%), 235 from individuals working in NHS trusts (response rate 40%) and 85 from individuals in independent hospitals (response rate 48%).

Most responses from independent hospitals came from hospital directors (41%), clinical governance leads (14%) and clinical leads (13%). For NHS trusts, most responses came from chief medical officers, medical directors and responsible officers (44%), clinical governance leads (21%) and senior nurse leaders (18%). NHS respondents were more likely to select “I don’t know” compared with respondents from independent hospitals, and the frequency of “I don’t know” responses varied across roles.

### Arrangements for shared clinical governance between NHS and independent hospitals

As Table 1 shows, most independent hospital respondents reported that the consultants working at their hospital come from multiple NHS hospitals/trusts, while NHS hospital respondents tended to say that their consultants who undertake independent practice mostly work at just one or two independent hospitals. NHS hospital respondents were also more likely to say they did not know which of their consultants worked in the independent sector, or what independent hospitals their consultants worked at.

**Table 1.** The number of NHS trusts and independent sector hospitals consultants come from/work at.

	One	Two	Three or more	I don’t know	Total
The number of independent sector hospitals the NHS respondents reported their consultants work at	69 (29.4)	53 (22.6)	32 (13.6)	81 (34.5)	235
The number of NHS trusts the independent sector respondents reported their consultants come from	27 (31.8)	16 (18.8)	39 (45.9)	3 (3.5)	85

Values are presented as frequency (percentage)

Both NHS and independent hospital respondents reported that they had some contact with the hospitals in the other sector, but only around 1 in 5 said they had a formal agreement or policy on shared arrangements for clinical governance. Both reported having meetings – often relatively ad hoc or infrequent. Independent hospital respondents reported more meetings and were more likely to say they were formal/minuted than NHS respondents. We asked about the arrangements for clinical governance between their hospitals and the other sector hospital which they work most closely (Table 2). Overall, independent hospital respondents rated the arrangements for shared clinical governance significantly more positively than those from the NHS (z = −3.16, p = .002).

**Table 2.** Ratings of the clinical governance arrangements between the sectors.

How would you describe the clinical governance arrangements you have with the other sector hospital?	Very good	Good	Acceptable	Poor	Very poor	I don’t know	Total
NHS	9 (13.0)	17 (24.6)	17 (24.6)	2 (2.9)	1 (1.5)	23 (33.3)	69
Independent sector	7 (22.6)	15 (48.4)	6 (19.4)	1 (3.2)	0 (0)	2 (6.5)	31

Values are presented as frequency (percentage)

We asked respondents to describe clinical governance arrangements with the other sector hospital, focusing on the hospital they work with the most. The quality of relationships described were varied, with some described as excellent, and others as poor or in need of improvement. Sometimes there was a strong relationship with one or two of the hospitals they worked with the most but little or no relationship with other hospitals with whom they share consultants. Many described the interaction between the sectors as sporadic and reactive, making contact when required in response to an issue or concern. Some relationships were reliant on individuals or personal connections and when these connections did not exist or were lost over time, for example due to staff changes, relationships were described as poor. RO to RO conversations were described as one of the main forms of communication between the sectors. Others depended on the probity of consultants to provide information about their work in the other sector.

Where there was a contractual agreement between the sectors for providing care, clinical governance was mainly managed by the NHS, with investigations carried out jointly. Some had seen improvements in relationships since the introduction of the Patient Safety Incident Response Framework (PSIRF), which outlines the NHS approach to responding to patient safety incidents in ways that support learning and improve patient safety.^36^ The COVID-19 pandemic was a catalyst for the formation of relationships between the sectors, but some suggested that this has not been maintained. Some described how relationships were maintained through the oversight of Integrated Care Boards (ICBs), statutory NHS organisations in England responsible for the planning, commissioning and funding of local health services, under the National Quality Board’s recommendations.

The two main private hospitals nearby, we have reasonable relationships. I made an effort as CMO to reach out to all local private hospitals, with varying degrees of success. It is all very ad hoc. I have had this conversation with the GMC ELA and RO meetings, that the flow of information and collaborative working needs to improve. (Chief Medical Officer, NHS)

Inconsistent - some are excellent and others less so. Regular meetings and shared learning happens between some hospitals and their local NHS trust. In some localities this is even broader with good relationships across all providers in the ICB region. Some hospitals really struggle to get good collaboration with the local NHS trust. (Chief Clinical Officer, Independent Sector)

### Sharing information about consultants’ scope of practice

Respondents described the process for obtaining and verifying supporting information about scope of practice. Descriptions of the arrangements for sharing such information about consultants’ scope of practice between sectors were quite variable. NHS respondents mostly commented this was done as part of annual appraisals, where a consultant would report on their independent practice and in some cases provide a “letter of good standing” from the independent hospital(s) where they worked. Independent hospital respondents largely said that consultants provided information on their NHS scope of practice when they were applying for practising privileges at the independent hospital, and this was sometimes supported by a letter or report from their NHS hospital, or by information from national registries (like the National Joint Registry for orthopaedic surgeons). Respondents from both sectors highlighted the limitations of the processes in place, such as the reliance on doctor probity, limited verification, lack of detail about procedures across sectors and lack of engagement on the part of the other sector.

At point of revalidation, we get a statement of no concerns from each IS [independent sector] organisation listed in scope of practice at appraisal. It does not tell us in detail if they do the same procedures as in the NHS. (RO, NHS)

I would write to the RO equivalent at the independent hospital if this is not clear in an appraisal process. Often a log is provided by a consultant though this does rely on probity. (Consultant Surgeon, NHS)

For some specialties the consultant is requested to get their scope of practice countersigned by their NHS Clinical Director. But compliance is variable - some NHS Clinical Directors are reluctant to engage. (Chief Executive, Independent Sector)

To be honest we get it from the consultant as part of PPs [practising privileges] and you are trusting the consultant that they are telling the truth. We probably don’t recheck scope of practice as much as we should.” (Hospital Director, Independent Sector)

Responses about the robustness of processes for verifying scope of practice are presented in Table 3. Nearly two thirds of respondents from independent hospitals either strongly agreed or agreed that their processes for verifying scope of practice were robust compared with less than a fifth of respondents from the NHS. Over a third of NHS leaders and a small number of independent sector leaders disagreed or strongly disagreed that their processes were robust. Also detailed in Table 3, three quarters of leaders from independent hospitals strongly agreed or agreed that they were confident they knew about the scope of practice of consultants who also work at the other sector hospital, compared with under a third of leaders from the NHS. Over a quarter of NHS leaders disagreed or strongly disagreed that they were confident.

**Table 3.** Agreement with how robust processes for verifying scope of practice are in their organisation and confidence about knowledge of scope of practice of consultants who also work in the other sector.

Questions	Sector	Strongly agree	Agree	Neither agree nor disagree	Disagree	Strongly disagree	I don’t know	Total
Our processes for verifying supporting information for scope of practice are robust	NHS	7 (4.0)	33 (18.6)	25 (14.1)	11 (6.2)	64 (36.2)	37 (20.9)	177
	Independent sector	21 (28.0)	31 (41.3)	3 (4.0)	0 (0)	6 (8.0)	14 (18.7)	75
I am confident that our organisation knows about the scope of practice of consultants who also work at the other sector hospita*l*	NHS	9 (5.1)	45 (25.6)	28 (15.9)	10 (5.7)	41 (23.3)	43 (24.4)	176
	Independent sector	23 (30.7)	35 (46.7)	2 (2.7)	0 (0)	3 (4.0)	12 (16.0)	75

Values are presented as frequency (percentage)

The arrangements for sharing information about scope of practice and respondents’ confidence in the information varied, with more concern expressed by NHS respondents. Respondents called for higher quality data, improved communication and information sharing and more robust, formalised and standardised processes. A number of suggestions for improvements were made including: mutual access to appraisal records; a centralised data repository which includes case numbers across a consultant’s NHS and independent practice; scope of practice statements with defined level of detail signed by all employers and the consultant; and improved systems and processes for the sharing of information in sufficient detail about consultant practice.

It would be ideal to have sufficient detail documented for all NHS consultants that could be shared with IS, and vice versa. Collecting, verifying, documenting and sharing this information would require systems and process we currently do not have. (Chief Medical Officer, NHS)

Automated letters of good standing sent in by the private provider would be really helpful. Also helpful would be for us to have a “go to” to private providers so that we can get up to date info about a doctors’ practice/ performance. (Hospital Director, NHS)

Improvement should involve a bidirectional interface - full NHS scope of practice can come from the Director of Service (DoS) for consultants and last 5 years of practical experience. This could be replicated by private providers. It is not just the scope of practice, rather the complications that are managed and clear understanding of outliers is useful. Our trust (trust name) uses a risk adjusted system (system name) to assess the observed and expected complications for each consultant every month. Data of this high quality (particularly if reciprocated) could lead to high quality assurance for patient care within both sectors and comply with the Paterson outcomes. (Assistant Medical Director, NHS)

A centralised data repository which includes case numbers across a consultant's NHS and private practice, which was the intention with PHIN (but it has not delivered yet). (Chief Executive, Independent Sector)

### Dealing with concerns about consultants’ practice

Over three quarters (79%) of respondents from NHS and nearly all respondents from the independent sector (93%) reported that their organisations had a policy on handling consultant concerns. Under two thirds (64%) of NHS respondents compared with nearly all independent sector respondents (93%) reported that the policy included a process for sharing information about consultant concerns with the other sector hospital(s).

Both NHS and independent hospital respondents said that concerns were shared through RO’s, medical directors or hospital directors – usually by email, phone or using the medical practice information transfer (MPIT) form (a form designed to support the appropriate transfer of information about a doctor’s practice to and from the doctor’s responsible officer).^37^ Respondents said that consultants do not necessarily inform other hospitals when there is a concern, problem, restriction on practice or other action involving them at a particular hospital.

As shown in Table 4, when asked about the sharing of significant concerns, interim restrictions on practice, substantive restrictions on practice, suspension/exclusion, and referrals to the GMC, both NHS and independent respondents indicated that these would often but not always be shared with hospitals in the other sector. They also reported hospitals in the other sector were much less likely to share such significant concerns with them. When there were cases involving the GMC about a consultant’s practice at another hospital, respondents were not confident that the GMC would always know that the consultant worked at their hospital and would inform them about the case.

**Table 4.** Frequency of sharing information from the respondent’s organisation to the other sector hospital regarding consultant restrictions, exclusion and referral and fulfilment of consultant professional obligation to inform other organisations about significant concerns, restrictions, exclusions/suspensions.

		Always	Often	Sometimes	Rarely	Never	I don’t know	Total
If there is a significant concern about one of your consultants, do you share information with the other sector hospital?	NHS	83 (47.7)	22 (12.6)	19 (10.9)	0 (0)	1 (0.6)	49 (28.2)	174
	Independent sector	67 (89.3)	3 (4.0)	2 (2.7)	0 (0)	0 (0)	3 (4.0)	75
If interim restrictions (temporary restrictions pending the outcome of an investigation) are placed on the practice of one of your consultants, do you share information with the other sector hospital?	NHS	96 (59.3)	12 (7.4)	8 (4.9)	1 (0.6)	1 (0.6)	44 (27.2)	162
	Independent sector	64 (85.3)	2 (2.7)	3 (4.0)	0 (0)	0 (0)	6 (8.0)	75
If substantial restrictions (restrictions based on conclusions from an investigation) are placed on the practice of one of your consultants, do you share information with the other sector hospital?	NHS	98 (60.9)	12 (7.5)	4 (2.5)	0 (0)	1 (0.6)	46 (28.6)	161
	Independent sector	65 (85.5)	4 (5.3)	2 (2.6)	0 (0)	0 (0)	5 (6.6)	76
If a consultant in your organisation is suspended/excluded for any reason, do you share information with the other sector hospital?	NHS	102 (63.4)	9 (5.6)	5 (3.1)	0 (0)	0 (0)	45 (28.0)	161
	Independent sector	69 (90.8)	3 (4.0)	1 (1.3)	0 (0)	0 (0)	3 (4.0)	76
If a consultant in your organisation is referred to the general medical council (GMC), do you share information with the other sector hospital?	NHS	72 (44.7)	19 (11.8)	20 (12.4)	2 (1.2)	1 (0.6)	47 (29.2)	161
	Independent sector	64 (84.2)	3 (4.0)	1 (1.3)	2 (2.6)	0 (0)	6 (8.0)	76
Do consultants fulfil their professional obligation to inform other organisations about significant concerns, restrictions, exclusions/suspensions (including the other sector hospital)?	NHS	20 (13.1)	46 (30.1)	17 (11.1)	1 (0.7)	0 (0)	69 (45.1)	153
	Independent sector	16 (21.1)	28 (36.8)	19 (25.0)	0 (0)	0 (0)	13 (17.1)	76

Values are presented as frequency (percentage)

Also shown in Table 4, we asked if consultants fulfil their professional obligation to inform other organisations about significant concerns, restrictions, exclusions/suspensions, including informing the other sector hospital they work in. Just less than half of NHS respondents did not know, but on the whole under a third of NHS and over a third of independent sector respondents felt that this often happened. Less than a quarter of independent sector respondents and more than a tenth of NHS respondents reported that this always happened.

We asked respondents to describe the arrangements for sharing information about concerns, restrictions and referrals between their organisation and the other sector hospital. Dependence on doctor probity for the sharing of information was highlighted as a potential concern; respondents only know what they were being told with no guarantees of full information. If doctors were not communicating relevant information about concerns, this might not get picked up if they were working in larger independent hospitals, where it may be easier for concerns to go unnoticed. Respondents felt that because the process for sharing concerns was non-mandatory and unregulated, it was under-resourced, and this made it harder to verify information. Strong relationships between the sectors resulted in effective communication but it was seen as a barrier when communication was dependent on these relationships and it also meant that a lot of communication was informal and undocumented. Both sectors stated that when there was not an established relationship it was not always clear who to contact. Independent sector respondents stated that it was often difficult to engage or get information from the NHS. Some felt it was not a mutual exchange of information, that they would provide details to the NHS trust but this would not always be reciprocated. Reasons included the challenges faced by the NHS, the size of NHS organisations and unclear lines of communication, knowledge of where independent work is happening, and communication with the independent sector not being prioritised. Sometimes requests for information would take time or be delayed or the information was just not passed on, resulting in delays in addressing or reviewing the problems.

I think the systems are reliant on personal honesty and transparency. If a consultant had practicing rights in [a large private hospital chain] for instance we may not know that. Locally the private hospitals are small and so it would be harder for folks to go under the radar. (Clinical Lead, NHS)

It is quite dependent on the individuals involved and the relationships they have. Our organisation has more than 30 hospitals, leading to conversations with RO’s in potentially many more trusts. It is difficult to build an effective relationship across this number. We therefore ask our Registered Managers to ensure they have a good relationship with the RO/medical staffing team in their local trust to try and ensure intelligence is shared. Many doctors do share information with us, but some do not. We also have a good relationship with our GMC liaison officer, who will keep us updated on aspects related to their investigations. (Chief Clinical Officer, Independent Sector)

It is not a two-way street - we inform but we are not often informed. This may be because it may be unknown where a consultant has their private practice as not held centrally anywhere (or validated). (VP of Quality, Independent Sector)

## Discussion

This paper presents the detailed findings from a national survey of senior leaders in NHS and independent acute hospitals in England about how clinical governance and quality and safety arrangements work between them. Overall, respondents identified several challenges related to clinical governance at the interface between the NHS and the independent sector. While some respondents acknowledged positive relationships with counterparts in the other sector and deemed the arrangements to be relatively sound, these connections frequently depended on personal goodwill and individual relationships rather than being established on systematic and consistent practices. There tended to be good relationships with specific hospitals from the opposing sector; however, relationships with other institutions in the vicinity were often less robust, leading to a heightened dependence on the integrity and probity of individual consultants. Additionally, respondents from both sectors indicated that the willingness of the “other sector” to share information and foster collaborative relationships was not always evident. NHS respondents were more likely to select “I don’t know” than those from independent hospitals, with variation across roles. This suggests that awareness of clinical governance arrangements may be influenced by organisational structure. In larger NHS organisations, where responsibilities for clinical governance are often divided across multiple functions (e.g. quality, safety, complaints), individuals may be less able to answer questions outside their specific role, compared with staff in smaller, more centrally led independent hospitals.

The results from this research highlight a continued reliance on doctor probity, with scepticism from both sectors that doctors provide all the necessary information about their practice or notify them in case of a concern. This reinforces the need for the implementation of the recommendations from the Paterson inquiry, particularly recommendation 1 which proposed a single repository of the whole practice of consultants across England, accessible to both sectors and the public, including practising privileges and consultant performance data; and recommendation 12b which proposed that if a healthcare professional also works at another provider, any concerns about them should be communicated to that provider.^2^ In regard to recommendation 1, since 2019, the Private Healthcare Information Network (PHIN), an independent, government-mandated organisation publishing performance and fees information about private consultants and hospitals, has been working with NHS Digital on ADAPt (the acute data alignment programme)^38^ to provide for the first time a combined data set covering all NHS and independent sector elective patient care. This was piloted during 2020-2023 but has not yet been implemented. The government’s response to recommendation 12b suggested that the Medical Profession (RO’s) Regulations 2010 (revised in 2013) allow for the sharing of information about performance concerns between health organisations, but we know from previous research on the implementation of medical revalidation in healthcare organisations in the UK, that the transfer of information about doctors’ performance between organisations is problematic.^39^

These findings indicate that governance at the NHS–independent sector interface relies heavily on informal processes, such as personal relationships and professional self-regulation, rather than the formal managerial governance structures increasingly adopted in healthcare over the past decades. This pattern reflects wider research showing that formal governance systems alone cannot ensure effective oversight; their success depends on how accountability processes interact with professional norms and leadership behaviours.^28^ Evidence from NHS boards highlights that engaged and transparent leadership is crucial for shaping culture and improving quality and safety.^29^ The reliance on informal relationships and professional probity seen in this study highlights the need for more systematic, standardised processes and stronger shared accountability across sectors. If these governance gaps are not addressed, there is a risk that both longstanding safety and learning systems, such as incident reporting, complaints and coroner investigations, and emergent initiatives, including value-based healthcare and the use of Patient-Reported Experience Measures (PREMs) and Patient-Reported Experience Measures (PROMs),^40^ may be compromised. The findings point to an urgent need for organisations to take definitive action, with timely implementation and monitoring of Paterson Inquiry recommendations critical to ensure safe, high-quality care.

### Strengths and limitations

This is the first national survey of senior leaders in both NHS and independent sector hospitals about clinical governance relationships across the sectors. Some respondents said that our research project provided much needed focus to these issues, welcoming change and reflective discussions on their clinical governance arrangements that survey questions prompted. It was felt that this could facilitate improvements in clinical governance practices.

The views and perceptions collected in this survey vary depending on both the representativeness of the respondents and their awareness of the topics covered. Medical Directors, RO’s and Chief Medical Officers would be expected to have a good understanding of clinical governance and relationships across the sectors, but respondents in other roles may not have sufficient experience and knowledge to answer all questions fully. This was a survey of individuals and not organisations, responses are the views of just one person in the organisation and may differ from the views of others in the same organisation. The survey focused specifically on senior healthcare leaders, as the nature of the questions required an overarching understanding of the clinical governance arrangements across the sectors, however further insights into how clinical governance arrangements work in practice and on the ground could be gained from doctors, nurses and other non-medical staff, and patients.

## Conclusions

We find that while there are some interorganisational clinical governance structures and relationships in place in England, there are significant concerns about how they work in practice. Both NHS and independent sector organisations should evaluate the effectiveness of their systems for sharing information about consultant concerns. Additionally, NHS organisations ought to ensure they know which consultants engage in independent practice and the independent hospitals where these consultants work. Regulatory bodies might consider revising national guidelines on shared clinical governance frameworks, taking into account the difficulties associated with information sharing between sectors, particularly due to incompatible IT systems and the reliance on paper records in smaller organisations. These findings may also be relevant to healthcare systems in other countries where clinicians work across multiple healthcare providers and there is a need for shared arrangements to ensure the quality and safety of patient care. There is a pressing need for further empirical research to compare clinical governance arrangements across sectors and to propose methods by which NHS and independent hospitals, along with entities such as Integrated Care Boards (ICBs), can establish shared clinical governance that ensures the quality and safety of patient care.

## Supplemental material

Supplemental material - Shared clinical governance arrangements between NHS and independent acute hospitals in England: Findings from a national survey of senior leaders Supplemental Material for Shared clinical governance arrangements between NHS and independent acute hospitals in England: Findings from a national survey of senior leaders by Gemma Stringer, Jane Ferguson, Kieran Walshe, Christos Grigoroglou, Thomas Allen, Michael Anderson, Karen Bloor, Eleanor Gee, Nils Gutacker in Journal of Health Services Research & Policy.

## Data Availability

The data that support the findings of this study are available from the corresponding author, [GS], upon reasonable request.*

## Abbreviations

A&E: Accident and Emergency
ADAPt: Acute Data Alignment Programme
CQC: Care Quality Commission
CMO: Chief Medical Officer
DoS: Director of Services
ELA: Employer Liaison Advisor
GMC: General Medical Council
HSIB: Health Services Safety Investigations Body
ICB: Integrated Care Board
IHPN: Independent Healthcare Provider Network
ITU: Intensive Therapy Unit
MPAF: Medical Practitioners Assurance Framework
MPIT: Medical Practice Information Transfer Form
NHS: National Health Service
NHSD: NHS Digital
PHIN: Private Healthcare Information Network
PREMs: Patient-Reported Experience Measures
PROMs: Patient-Reported Experience Measures
PSIRF: Patient Safety Incident Response Framework
RO: Responsible Officer
