# Supplementary material for: Shared clinical governance arrangements between NHS and independent acute hospitals in England: Findings from a national survey of senior leaders
Source: J Health Serv Res Policy. 2026 Mar 19;31(4):234–44. doi: 10.1177/13558196261431315 (PMC13589108; doi:10.1177/13558196261431315)
Supplement: Supplementary file 1 [file sj-pdf-1-hsr-10.1177_13558196261431315.pdf]

Online Supplement: Survey

National survey of shared clinical governance across the NHS and independent hospitals in England

---

Start of Block: Introduction

National survey of shared clinical governance across NHS and independent acute hospitals in England

This is a survey about the effectiveness of shared arrangements for clinical governance between NHS and acute independent hospitals and has been funded by the National Institute for Health and Care Research.

The findings of this survey will provide, for the first time, a comprehensive dataset on how clinical governance arrangements work between the NHS and independent hospitals. By completing this survey, you will contribute to a better understanding of the quality and safety of patient care in NHS and independent hospitals.

We are committed to using findings from this survey to inform and improve clinical governance between NHS and independent hospitals. Your contributions will help inform future decisions about how clinical governance is organised across sectors and provide important information to improve patient safety. We are asking people involved in clinical governance from NHS and independent hospitals, such as medical directors, chief nurses and clinical governance leads, to complete the survey. We are interested in views about clinical governance processes between NHS hospitals and independent hospitals including: whole scope of practice appraisal and revalidation, dealing with significant concerns, understanding capacity and capability, sharing patient information for elective care and transfers and the impact of the Covid 19 pandemic.

Participant information

Please follow this link to read the full participant information: Participant Information Sheet.

Ethical approval

The project has been reviewed by NHS REC (23/EE/0104) and has been given HRA approval.

Statement of Confidentiality

Everything you say in this questionnaire will remain confidential. Any information that would permit identification of an individual or an organisation will be strictly confidential, will be used only for the purposes of this study, and will not be disclosed or released to other persons/organisations or used for any other purpose.

After your responses have been entered onto a secure computer database, the survey will be securely stored in accordance with data protection regulations and destroyed after five years.

If you would like to know more about the study, please contact:

[removed for anonymised peer review process]

The questionnaire will take around 15 to 20 minutes to complete. You can pause any time you like and finish the questionnaire later. By filling in the survey, you are indicating that you have read the information provided and agree to participate.

We ask that you try and answer all the questions you can, but if there are questions that you cannot find the information to answer or it is out of the remit of your role then you have the option to answer "don't know" or to leave the question blank.

If you consent to take part in the survey then please tick below and then click on the arrow to start the survey.

○ I consent to take part in the survey (1)

End of Block: Introduction

Start of Block: About you – role, experience and responsibilities for clinical governance

We would like to ask you some questions about yourself, where you work and your job role.

Where do you work?

- ◯ NHS (1)
- ◯ Independent sector (2)
- ◯ Both (NHS and independent sector) (3)

Display This Question:

If Where do you work? = Both (NHS and independent sector)

Where do you **mainly** work?

- ◯ NHS (1)
- ◯ Independent sector (2)

What is your current **main** job role?

- ◯ Chief Executive (1)
- ◯ Chief Medical Officer (2)
- ◯ Chief Nurse (3)
- ◯ Clinical Governance Lead (4)
- ◯ Director of Clinical Services (5)
- ◯ Director of Nursing (6)
- ◯ Hospital Director (7)
- ◯ Matron (8)
- ◯ Medical Director (9)
- ◯ Responsible Officer (10)
- ◯ Other [please write in] (11) ____

Display This Question:

If Where do you work? = Independent sector

Or Where do you mainly work? = Independent sector

Does your role relate to an individual hospital or a group of hospitals?

- ◯ Individual hospital (1)
- ◯ Group of hospitals (2)

Do you have another job role/s?

- Yes (1)
- No (2)

Display This Question:

If Do you have another job role/s? = Yes

Please select your other job role/s [please select all that apply]

- Chief Executive (1)
- Chief Medical Officer (2)
- Chief Nurse (3)
- Clinical Governance Lead (4)
- Director of Clinical Services (5)
- Director of Nursing (6)
- Hospital Director (7)
- Matron (8)
- Medical Director (9)
- Responsible Officer (10)
- Other [please write in] (11) ____

Display This Question:

If Where do you work? = NHS

Have you previously worked in an independent hospital?

- Yes (1)
- No (2)

Display This Question:

If Where do you work? = Independent sector

Have you previously worked in an NHS hospital?

- Yes (1)
- No (2)

End of Block: About you – role, experience and responsibilities for clinical governance

Start of Block: About your organisation and relationships - NHS

We would like to ask you about your relationships with hospitals in the independent sector where consultants from your organisation also work.

Which of the following best describes the consultants that work at your organisation who also work in the independent sector?

- Our consultants mainly work at one independent hospital (1)
- Our consultants mainly work at two independent hospitals (2)
- Our consultants mainly work at three or more independent hospitals (3)
- I don't know (4)

Thinking about the independent hospitals where your consultants also work.

How many of the independent hospitals do you have contact with?

- ◯ We have contact with all of the independent hospitals (1)
- ◯ We have contact with most of the independent hospitals (2)
- ◯ We have contact with some of the independent hospitals (3)
- ◯ We have contact with none of the independent hospitals (4)
- ◯ I don't know (5)

Skip To: End of Block If Thinking about the independent hospitals where your consultants also work. How many of the indepe... = We have contact with none of the independent hospitals

Display This Question:

If Thinking about the independent hospitals where your consultants also work. How many of the indepe... != We have contact with none of the independent hospitals

Of the hospitals you have contact with please tell us about what your clinical governance relationships are like [please write in].

____

End of Block: About your organisation and relationships - NHS

Start of Block: Clinical governance processes - NHS

We understand that consultant medical staff in your organisation might work across multiple organisations (e.g., NHS and different independent hospitals). We would like you to answer the following questions in relation to ONE organisation. What we mean by this is the independent hospital where the largest number of your consultant medical staff also work.

Thinking about the independent hospital where the largest number of your consultant medical staff also work, we would like to ask about the clinical governance processes between your organisation and the independent hospital.

Do you have a formal agreement or policy in place with the independent hospital on how you collaborate on clinical governance processes?

- Yes (1)
- No (2)
- I don't know (3)

Do you meet with the independent hospital about clinical governance?

- Yes (1)
- No (2)
- I don't know (3)

Skip To: End of Block If Do you meet with the independent hospital about clinical governance? = No

---

On average, how often do you meet with staff responsible for clinical governance from the independent hospital?

- Once a week (1)
- Once a month (2)
- Once every 3 months (3)
- Once every 6 months (4)
- Once a year (5)
- Less than once a year (6)
- Never (7)
- I don't know (8)

Skip To: End of Block If On average, how often do you meet with staff responsible for clinical governance from the indepen... = Never

---

Are these meetings formal (e.g., minuted, contractually required) or informal (e.g., ad hoc)?

- Formal (1)
- Informal (2)
- Both formal and informal (3)
- I don't know (4)

Please provide details of what happens at meetings (e.g., are any reports submitted or are there contracts with quality requirements) [please write in]

____

How would you describe the clinical governance arrangements you have with the independent hospital?

- Very good (1)
- Good (2)
- Acceptable (3)
- Poor (4)
- Very poor (5)
- I don't know (6)

Do you share learning from patient safety incidents with the independent hospital?

- Always (1)
- Often (2)
- Sometimes (3)
- Rarely (4)
- Never (5)
- I don't know (6)

How have your clinical governance arrangements with the independent hospital developed? [please write in]

____

How could the clinical governance relationship with the independent hospital be improved? [please write in]

____

End of Block: Clinical governance processes - NHS

Start of Block: Scope of practice, appraisal and revalidation - NHS

Do you know which of your consultants are also working at the independent hospital?

○ I know this for all of the consultants (1)

○ I know this for most of the consultants (2)

○ I know this for some of the consultants (3)

○ I do not know this for any of the consultants (4)

Thinking about your consultants who also work at the independent hospital, we want to ask you about scope of practice.

Does your organisation get supporting information about the scope of practice for consultants who also work at the independent hospital?

○ Always (1)

○ Often (2)

○ Sometimes (3)

○ Rarely (4)

○ Never (5)

○ I don't know (6)

What is the process for getting supporting information about scope of practice? [please write in]

____

How is scope of practice information verified? [please write in]

____

Our processes for verifying supporting information for scope of practice are robust.

- ○ Strongly agree (1)
- ○ Agree (2)
- ○ Neither agree nor disagree (6)
- ○ Disagree (3)
- ○ Strongly disagree (4)
- ○ I don't know (5)

I am confident that our organisation knows about the scope of practice of consultants who also work at the independent hospital.

- ◯ Strongly agree (1)
- ◯ Agree (2)
- ◯ Neither agree nor disagree (6)
- ◯ Disagree (3)
- ◯ Strongly disagree (4)
- ◯ I don't know (5)

Are revalidation decisions delayed by not having supporting information about consultants' practice in independent hospitals?

- ◯ Always (1)
- ◯ Often (2)
- ◯ Sometimes (3)
- ◯ Rarely (4)
- ◯ Never (5)
- ◯ I don't know (6)

Please tell us about the arrangements for verifying scope of practice information for your consultants who also work at the independent hospital and how this could be improved. [please write in]

____

End of Block: Scope of practice, appraisal and revalidation - NHS

Start of Block: Identifying and dealing with concerns/complaints - NHS

We would like to ask you about sharing information about the practice of your consultants who also work at the independent hospital.

Does your organisation have a policy on handling consultant concerns?

- ○ Yes (1)
- ○ No (2)
- ○ I don't know (3)

Display This Question:

If Does your organisation have a policy on handling consultant concerns? = Yes

Does this include a process for sharing information about concerns with the independent hospital?

- ○ Yes [if yes, please provide further details] (1) ____
- ○ No (2)
- ○ I don't know (3)

We would like to ask how you share information with the independent hospital and how they share information with you.

Thinking about your consultants, who also work at the independent hospital, we would like to ask you about sharing information about significant concerns, restrictions, suspensions/exclusions or referrals.

When we refer to a significant concern we mean a concern that results in an investigation.

If there is a significant concern about one of your consultants, do you share information with the independent hospital?

- Always (1)
- Often (2)
- Sometimes (3)
- Rarely (4)
- Never (5)
- I don't know (6)

Display This Question:

If If there is a significant concern about one of your consultants, do you share information with th... != Never

When do you share information about significant concerns? [please select all that apply]

- Before we have started investigating (1)
- When we are investigating (2)
- After we have investigated (3)
- At multiple stages during investigation (4)
- I don't know (5)

If there is a significant concern about one of your consultants at the independent hospital, do they share this information with you?

- ○ Always (1)
- ○ Often (2)
- ○ Sometimes (3)
- ○ Rarely (4)
- ○ Never (5)
- ○ I don't know (6)

If interim restrictions (temporary restrictions pending the outcome of an investigation) are placed on the practice of one of your consultants, do you share information with the independent hospital?

- ○ Always (1)
- ○ Often (2)
- ○ Sometimes (3)
- ○ Rarely (4)
- ○ Never (5)
- ○ I don't know (6)

If the independent hospital places interim restrictions on the practice of one of your consultants, do they share this information with you?

- Always (1)
- Often (2)
- Sometimes (3)
- Rarely (4)
- Never (5)
- I don't know (6)

If substantial restrictions (restrictions based on conclusions from an investigation) are placed on the practice of one of your consultants, do you share information with the independent hospital?

- Always (1)
- Often (2)
- Sometimes (3)
- Rarely (4)
- Never (5)
- I don't know (6)

If the independent hospital places substantial restrictions on the practice of one of your consultants, do they share this information with you?

- Always (1)
- Often (2)
- Sometimes (3)
- Rarely (4)
- Never (5)
- I don't know (6)

Page Break

If a consultant in your organisation is suspended/excluded for any reason, do you share information with the independent hospital?

- Always (1)
- Often (2)
- Sometimes (3)
- Rarely (4)
- Never (5)
- I don't know (6)

If a consultant at the independent hospital is suspended/excluded for any reason, do they share this information with you?

- Always (1)
- Often (2)
- Sometimes (3)
- Rarely (4)
- Never (5)
- I don't know (6)

If a consultant in your organisation is referred to the General Medical Council (GMC), do you share information with the independent hospital?

- Always (1)
- Often (2)
- Sometimes (3)
- Rarely (4)
- Never (5)
- I don't know (6)

If the independent hospital refers one of your consultants to the GMC, do they share this information with you?

- Always (1)
- Often (2)
- Sometimes (3)
- Rarely (4)
- Never (5)
- I don't know (6)

If a GMC referral is made by the independent hospital, does the GMC inform your hospital directly?

- ◯ Always (1)
- ◯ Often (2)
- ◯ Sometimes (3)
- ◯ Rarely (4)
- ◯ Never (5)
- ◯ I don't know (6)

If the GMC restricts the practice or suspends the licence of a consultant from the independent hospital, does the GMC inform you directly?

- ◯ Always (1)
- ◯ Often (2)
- ◯ Sometimes (3)
- ◯ Rarely (4)
- ◯ Never (5)
- ◯ I don't know (6)

Page Break

We want to ask you about sharing information regarding significant concerns, restrictions, exclusions/suspensions or GMC referrals about any of your consultants who also work at the independent hospital.

Who do you share information with at the independent hospital? [please select all that apply]

- ☐ Chief Executive (1)
- ☐ Chief Medical Officer (2)
- ☐ Chief Nurse (3)
- ☐ Clinical Governance Lead (4)
- ☐ Director of Clinical Services (5)
- ☐ Director of Nursing (6)
- ☐ Hospital Director (7)
- ☐ Matron (8)
- ☐ Medical Director (9)
- ☐ Responsible Officer (10)
- ☐ Other [please write in] (11)

____

What is the main method you use to share information with the independent hospital?

- ○ Medical Practice Information Transfer (MPIT) (1)
- ○ Telephone (2)
- ○ Email (3)
- ○ Other [please write in] (4) ____
- ○ I don't know (5)

Do consultants fulfil their professional obligation to inform other organisations about significant concerns, restrictions, exclusions/suspensions (including the independent hospital)?

- ○ Always (1)
- ○ Often (2)
- ○ Sometimes (3)
- ○ Rarely (4)
- ○ Never (5)
- ○ I don't know (6)

What are the strengths and what are the barriers with sharing information about concerns, restrictions and referrals between your organisation and the independent hospital? [please write in]

____

End of Block: Identifying and dealing with concerns/complaints - NHS

Start of Block: Understanding capacity and capability and patient information transfers - NHS

We want to ask you about your understanding of the independent hospital's capacity and capability.

How would you describe your knowledge about the capacity and capability of the independent hospital to treat deteriorating patients that require an escalation in care?

My knowledge about the capacity and capability of the independent hospital to treat deteriorating patients that require an escalation in care is...

- ○ Very good (1)
- ○ Good (2)
- ○ Acceptable (3)
- ○ Poor (4)
- ○ Very poor (5)
- ○ I don't know (6)

Which of the following do you know about the independent hospital? [please select all that apply]

- ☐ How many inpatient beds they have (1)
- ☐ How many operating theatres they have (2)
- ☐ Whether they have their own critical care facilities (3)
- ☐ What out of hours medical cover they have (4)
- ☐ None of these (5)

Page Break

We want to ask you about patient transfers and how information about patients is shared for clinical care.

Is there any form of written policy or agreement in place with the independent hospital on how you share or transfer patient clinical information and records?

- ○ Yes (1)
- ○ No (2)
- ○ I don't know (3)

When a patient is treated on an NHS-funded elective care pathway in the independent hospital, how is patient information transferred from your hospital? [please write in]

____

When there is an urgent transfer of a patient from the independent hospital to your organisation, do you receive timely and adequate information on the patient from the independent hospital?

- ○ Always (1)
- ○ Often (2)
- ○ Sometimes (3)
- ○ Rarely (4)
- ○ Never (5)
- ○ I don't know (6)

When there is an urgent transfer of a patient from the independent hospital to your organisation, do you provide information to the independent hospital about subsequent treatment and outcomes?

- ◯ Always (1)
- ◯ Often (2)
- ◯ Sometimes (3)
- ◯ Rarely (4)
- ◯ Never (5)
- ◯ I don't know (6)

What screening and risk management processes do you have in place for the safe transfer of patients from the independent hospital to your organisation? [please write in]

____

Please tell us about the arrangements for **sharing patient information** and **managing transfers** between your organisation and the independent hospital and how this could be improved? [please write in]

____

End of Block: Understanding capacity and capability and patient information transfers - NHS

Start of Block: Changes to clinical governance processes as a result of the pandemic - NHS

We want to ask you about changes to clinical governance processes as a result of the COVID19 pandemic.

During the COVID19 pandemic when independent hospitals were providing more NHS funded elective care, did the arrangements for clinical governance between your organisation and the independent hospital change?

- Yes (1)
- No (2)
- I don't know (3)

Display This Question:

If During the COVID19 pandemic when independent hospitals were providing more NHS funded elective ca... = Yes

What changes were there to the arrangements for clinical governance between your organisation and the independent hospital? [please write in]

____

Display This Question:

If During the COVID19 pandemic when independent hospitals were providing more NHS funded elective ca... = Yes

Are these changes still in place?

- Yes (1)
- No (2)
- I don't know (3)

Display This Question:

If Are these changes still in place? = Yes

What impact have the changes had on clinical governance arrangements between your organisation and the independent hospital?

____

End of Block: Changes to clinical governance processes as a result of the pandemic - NHS

Start of Block: Anything else - NHS

Is there anything else you would like to tell us about the clinical governance arrangements with the independent hospital?

____

End of Block: Anything else - NHS

Start of Block: About your organisation and relationships - independent sector

We would like to ask you about your relationships with hospitals in the NHS where consultants from your organisation also work.

Which of the following best describes the consultants that work at your organisation who also work in the NHS?

- ○ Our consultants mainly come from one NHS hospital/trust (1)
- ○ Our consultants mainly come from two NHS hospitals/trusts (2)
- ○ Our consultants mainly come from three or more NHS hospitals/trusts (3)
- ○ I don't know (4)

Thinking about the NHS hospitals where your consultants also work:

How many of the NHS hospitals do you have contact with?

- ○ We have contact with all of the NHS hospitals (1)
- ○ We have contact with most of the NHS hospitals (2)
- ○ We have contact with some of the NHS hospitals (3)
- ○ We have contact with none of the NHS hospitals (4)
- ○ I don't know (5)

Skip To: End of Block If Thinking about the NHS hospitals where your consultants also work: How many of the NHS hospitals... = We have contact with none of the NHS hospitals

Of the hospitals you have contact with please tell us about what your clinical governance relationships are like [please write in]

____

End of Block: About your organisation and relationships - independent sector

Start of Block: Clinical governance processes - independent sector

We understand that consultant medical staff in your organisation might work across multiple organisations (e.g., NHS and different independent hospitals). We would like you to answer the following questions in relation to ONE organisation. What we mean by this is the NHS hospital where the largest number of your consultant medical staff also work.

Thinking about the NHS hospital where the largest number of your consultant medical staff also work, we would like to ask about the clinical governance processes between your organisation and the NHS hospital.

Do you have a formal agreement or policy in place with the NHS hospital on how you collaborate on clinical governance?

- ○ Yes (1)
- ○ No (2)
- ○ I don't know (3)

Do you meet with the NHS hospital about clinical governance?

- ○ Yes (1)
- ○ No (2)
- ○ I don't know (3)

Skip To: End of Block If Do you meet with the NHS hospital about clinical governance? = No

---

On average, how often do you meet with staff responsible for clinical governance from the NHS hospital?

- Once a week (1)
- Once a month (2)
- Once every 3 months (3)
- Once every 6 months (4)
- Once a year (5)
- Less than once a year (6)
- Never (7)
- I don't know (8)

Skip To: End of Block If On average, how often do you meet with staff responsible for clinical governance from the NHS hos... = Never

Are these meetings formal (e.g., minuted, contractually required) or informal (e.g., ad hoc)?

- Formal (1)
- Informal (2)
- Both formal and informal (3)
- I don't know (4)

Please provide details of what happens at meetings (e.g., are any reports submitted or are there contracts with quality requirements) [please write in]

____

How would you describe the clinical governance arrangements you have with the NHS hospital?

- ○ Very good (1)
- ○ Good (2)
- ○ Acceptable (3)
- ○ Poor (4)
- ○ Very poor (5)
- ○ I don't know (6)

Do you share learning from patient safety incidents with the NHS hospital?

- ○ Always (1)
- ○ Often (2)
- ○ Sometimes (3)
- ○ Rarely (4)
- ○ Never (5)
- ○ I don't know (6)

How have your clinical governance arrangements with the NHS hospital developed? [please write in]

____

How could the clinical governance relationship with the NHS hospital be improved? [please write in]

____

End of Block: Clinical governance processes - independent sector

---

Start of Block: Scope of practice, appraisal and revalidation - independent sector

Do you know which of your consultants are also working at the NHS hospital?

- ◯ I know this for all of the consultants (1)
- ◯ I know this for most of the consultants (2)
- ◯ I know this for some of the consultants (3)
- ◯ I do not know this for any of the consultants (4)

Thinking about your consultants who also work in the NHS hospital, we want to ask you about scope of practice.

Does your hospital get supporting information about the scope of practice for consultants who also work at the NHS hospital?

- ◯ Always (1)
- ◯ Often (2)
- ◯ Sometimes (3)
- ◯ Rarely (4)
- ◯ Never (5)
- ◯ I don't know (6)

What is the process for getting supporting information about scope of practice? [please write in]

____

How is scope of practice information verified? [please write in]

____

Our processes for verifying supporting information for scope of practice are robust.

- ○ Strongly agree (1)
- ○ Agree (2)
- ○ Neither agree nor disagree (6)
- ○ Disagree (3)
- ○ Strongly disagree (4)
- ○ I don't know (5)

I am confident that our organisation knows about the scope of practice of consultants who also work at the NHS hospital.

- Strongly agree (1)
- Agree (2)
- Neither agree nor disagree (6)
- Disagree (3)
- Strongly disagree (4)
- I don't know (5)

How often do consultants share information about their appraisals done by the NHS hospital?

- Always (1)
- Often (2)
- Sometimes (3)
- Rarely (4)
- Never (5)
- I don't know (6)

Please tell us about the arrangements for verifying scope of practice information for your consultants who also work at the NHS hospital and how this could be improved. [please write in]

____

End of Block: Scope of practice, appraisal and revalidation - independent sector

---

Start of Block: Identifying and dealing with concerns/complaints - independent sector

We would like to ask you about sharing information about the practice of your consultants who also work at the NHS hospital.

Does your organisation have a policy on handling consultant concerns?

- ○ Yes (1)
- ○ No (2)
- ○ I don't know (3)

Display This Question:

If Does your organisation have a policy on handling consultant concerns? = Yes

Does this include a process for sharing information about concerns with the NHS hospital?

- ○ Yes [if yes, please provide further details] (1) ____
- ○ No (2)
- ○ I don't know (3)

We would like to ask about how you share information with the NHS hospital and how they share information with you.

Thinking about your consultants who also work at the NHS hospital, we would like to ask you about sharing information about significant concerns, restrictions, suspensions/exclusions or referrals.

When we refer to a significant concern we mean a concern that results in an investigation.

If there is a significant concern about one of your consultants, do you share information with the NHS hospital?

- Always (1)
- Often (2)
- Sometimes (3)
- Rarely (4)
- Never (5)
- I don't know (6)

Display This Question:

If If there is a significant concern about one of your consultants, do you share information with th... != Never

When do you share information about significant concerns?

- Before we have started investigating (1)
- When are investigating (2)
- After we have investigated (3)
- At multiple stages during investigation (4)
- I don't know (5)

If there is a significant concern about one of your consultants at the NHS hospital, do they share this information with you?

- ◯ Always (1)
- ◯ Often (2)
- ◯ Sometimes (3)
- ◯ Rarely (4)
- ◯ Never (5)
- ◯ I don't know (6)

If interim restrictions (temporary restrictions pending the outcome of an investigation) are placed on the practice of one of your consultants, do you share information with the NHS hospital?

- ◯ Always (1)
- ◯ Often (2)
- ◯ Sometimes (3)
- ◯ Rarely (4)
- ◯ Never (5)
- ◯ I don't know (6)

If the NHS hospital places interim restrictions on the practice of one of your consultants, do they share this information with you?

- Always (1)
- Often (2)
- Sometimes (3)
- Rarely (4)
- Never (5)
- I don't know (6)

If substantial restrictions (restrictions based on conclusions from an investigation) are placed on the practice of one of your consultants, do you share information with the NHS hospital?

- Always (1)
- Often (2)
- Sometimes (3)
- Rarely (4)
- Never (5)
- I don't know (6)

If the NHS hospital places substantial restrictions on the practice of one of your consultants, do they share this information with you?

- Always (1)
- Often (2)
- Sometimes (3)
- Rarely (4)
- Never (5)
- I don't know (6)

Page Break

If a consultant in your organisation is suspended/excluded for any reason, do you share information with the NHS hospital?

- Always (1)
- Often (2)
- Sometimes (3)
- Rarely (4)
- Never (5)
- I don't know (6)

If a consultant at the NHS hospital is suspended/excluded for any reason, do they share this information with you?

- Always (1)
- Often (2)
- Sometimes (3)
- Rarely (4)
- Never (5)
- I don't know (6)

If a consultant in your organisation is referred to the General Medical Council (GMC), do you share information with the NHS hospital?

- Always (1)
- Often (2)
- Sometimes (3)
- Rarely (4)
- Never (5)
- I don't know (6)

If the NHS hospital refers one of your consultants to the GMC, do they share this information with you?

- Always (1)
- Often (2)
- Sometimes (3)
- Rarely (4)
- Never (5)
- I don't know (6)

If a GMC referral is made by the NHS hospital, does the GMC inform your organisation directly?

- ○ Always (1)
- ○ Often (2)
- ○ Sometimes (3)
- ○ Rarely (4)
- ○ Never (5)
- ○ I don't know (6)

If the GMC restricts the practice or suspends the licence of a consultant from the NHS hospital, does the GMC inform you directly?

- ○ Always (1)
- ○ Often (2)
- ○ Sometimes (3)
- ○ Rarely (4)
- ○ Never (5)
- ○ I don't know (6)

Page Break

We want to ask you about sharing information regarding significant concerns, restrictions, exclusions/suspensions or GMC referrals about any of your consultants who also work at the NHS hospital.

Who do you share information with at the NHS hospital? [please select all that apply]

- ☐ Chief Executive (1)
- ☐ Chief Medical Officer (2)
- ☐ Chief Nurse (3)
- ☐ Clinical Governance Lead (4)
- ☐ Director of Clinical Services (5)
- ☐ Director of Nursing (6)
- ☐ Hospital Director (7)
- ☐ Matron (8)
- ☐ Medical Director (9)
- ☐ Responsible Officer (10)
- ☐ Other [please write in] (11) ____

What is the main method you use to share information with the NHS hospital?

- ◯ Medical Practice Information Transfer (MPIT) (1)
- ◯ Telephone (2)
- ◯ Email (3)
- ◯ Other [please write in] (4) ____
- ◯ I don't know (5)

Do consultants fulfil their professional obligation to inform other organisations about significant concerns, restrictions, exclusions/suspensions (including the NHS hospital)?

- ◯ Always (1)
- ◯ Often (2)
- ◯ Sometimes (3)
- ◯ Rarely (4)
- ◯ Never (5)
- ◯ I don't know (6)

What are the strengths and what are the barriers with sharing information about concerns, restrictions and referrals between your organisation and the NHS hospital? [please write in]

____

End of Block: Identifying and dealing with concerns/complaints - independent sector

---

Start of Block: Understanding capacity and capability and patient information transfers - independent sector

Display This Question:

If Does your role relate to an individual hospital or a group of hospitals? = Individual hospital

We want to ask you about the NHS hospitals understanding of your hospital's capacity and capability.

Display This Question:

If Does your role relate to an individual hospital or a group of hospitals? = Individual hospital

How would you describe the NHS hospitals knowledge about the capacity and capability of your hospital to treat deteriorating patients that require an escalation in care?

The NHS hospitals knowledge about the capacity and capability of the independent hospital to treat deteriorating patients that require an escalation in care is…

- ◯ Very good (1)
- ◯ Good (2)
- ◯ Acceptable (3)
- ◯ Poor (4)
- ◯ Very poor (5)
- ◯ I don't know (6)

Display This Question:

If Does your role relate to an individual hospital or a group of hospitals? = Individual hospital

Which of the following do you think the NHS hospital knows about the capacity and capability of your hospital? [please select all that apply]

- ☐ How many inpatient beds you have (1)
- ☐ How many operating theatres you have (2)
- ☐ Whether you have your own critical care facilities (3)
- ☐ What out of hours medical cover you have (4)
- ☐ None of these (5)

We want to ask you about patient transfers and how information about patients is shared for clinical care.

Is there any form of written policy or agreement in place with the NHS hospital on how you share or transfer patient clinical information and records?

- ○ Yes (1)
- ○ No (2)
- ○ I don't know (3)

When a patient is treated on an NHS-funded elective care pathway in your organisation, how is patient information transferred from the NHS hospital? [please write in]

____

When there is an urgent transfer of a patient from your organisation to the NHS hospital, do you send timely and adequate information about the patient to the NHS hospital?

- ◯ Always (1)
- ◯ Often (2)
- ◯ Sometimes (3)
- ◯ Rarely (4)
- ◯ Never (5)
- ◯ I don't know (6)

When there is an urgent transfer of a patient from your organisation to the NHS hospital, are you able to find out about their subsequent treatment and outcomes?

- ◯ Always (1)
- ◯ Often (2)
- ◯ Sometimes (3)
- ◯ Rarely (4)
- ◯ Never (5)
- ◯ I don't know (6)

What screening and risk management processes do you have in place for the safe transfer of patients to the NHS hospital? [please write in]

____

Please tell us about the arrangements for **sharing patient information** and **managing transfers** between your organisation and NHS hospital and how this could be improved? [please write in]

____

End of Block: Understanding capacity and capability and patient information transfers - independent sector

Start of Block: Changes to clinical governance processes as a result of the pandemic - independent sector

We want to ask you about changes to clinical governance processes as a result of the COVID19 pandemic.

During the COVID19 pandemic when independent hospitals were providing more NHS funded elective care, did the arrangements for clinical governance between your organisation and the NHS hospital change?

- Yes (1)
- No (2)
- I don't know (3)

Display This Question:

If During the COVID19 pandemic when independent hospitals were providing more NHS funded elective ca... = Yes

What changes were there to the arrangements for clinical governance between your organisation and the NHS hospital? [please write in]

____

Display This Question:

If During the COVID19 pandemic when independent hospitals were providing more NHS funded elective ca... = Yes

Are these changes still in place?

◯ Yes (1)

◯ No (2)

◯ I don't know (3)

Display This Question:

If Are these changes still in place? = Yes

What impact have the changes had on clinical governance arrangements between your organisation and the NHS hospital? [please write in]

____

End of Block: Changes to clinical governance processes as a result of the pandemic - independent sector

Start of Block: Anything else - independent sector

Is there anything else you would like to tell us about the clinical governance arrangements with the NHS hospital?

____

End of Block: Anything else - independent sector

Start of Block: About you - demographics

Please tell us about yourself.

What is your gender?

- ○ Woman (1)
- ○ Man (2)
- ○ Transgender woman (3)
- ○ Transgender man (4)
- ○ Non-binary/non-conforming (5)
- ○ Prefer not to respond (6)
- ○ Prefer to self describe as (7) ____

How old are you?

- ○ 18-24 years old (1)
- ○ 25-34 years old (2)
- ○ 35-44 years old (3)
- ○ 45-54 years old (4)
- ○ 55-64 years old (5)
- ○ 65+ years old (6)
- ○ Prefer not to respond (7)

What is your ethnic group or background?

Asian/Asian British

- [ ] Indian (1)
- [ ] Pakistani (2)
- [ ] Bangladeshi (3)
- [ ] Chinese (4)
- [ ] Any other Asian background [please describe] (5)

____

Black/Black British

- [ ] Caribbean (1)
- [ ] African (2)
- [ ] Any other Black/African/Caribbean background [please describe] (3)

____

Mixed/Multiple ethnic groups

- [ ] White & Black Caribbean (1)
- [ ] White & Black African (2)
- [ ] White & Asian (3)
- [ ] Any other mixed/multiple ethnic background [please describe] (4)

____

White

- ☐ English (1)
- ☐ Welsh (2)
- ☐ Scottish (3)
- ☐ Irish (4)
- ☐ Gypsy or Irish Traveller (5)
- ☐ Any other White background [please describe] (6) ____

Other ethnic group

- ☐ Arab (1)
- ☐ Any other ethnic group [please describe] (2) ____

End of Block: About you - demographics

Start of Block: Copy of the survey report

If you would like a copy of the survey report then please provide your email address below. Please be assured this information will be confidential and your email address will not be linked to any of your answers.

____

End of Block: Copy of the survey report

Online Supplement: Tables

Table S1. Respondent job roles in the NHS and independent sector

	Independent hospitals	NHS trusts
Executive role	8	7
Chief Medical Officer, Medical Director, Responsible Officer	9	104
Senior Nurse Leader	9	45
Clinical Governance Lead	12	52
Clinical Lead	11	10
Hospital Director	35	0
Consultant	0	12
Other	1	5
**Total**	**85**	**235**

Table S2. Where consultants from the NHS and independent sector also work or come from in the other sector

Which of the following best describes the consultants that work at your organisation who also work in the other sector?	Our consultants mainly work at one hospital/come from one trust	Our consultants mainly work at two hospitals/come from two trusts	Our consultants mainly work at three or more hospitals/come from three or more trusts	I don't know	Total
**NHS**	69 (29.4)	53 (22.6)	32 (13.6)	81 (34.5)	235
**Independent sector**	27 (31.8)	16 (18.8)	39 (45.9)	3 (3.5)	85

Table S3. Contact between the NHS trusts and the independent hospitals

How many of the hospitals from the other sector do you have contact with?	**We have contact with all of the hospitals**	**We have contact with most of the hospitals**	**We have contact with some of the hospitals**	**We have contact with none of the hospitals**	**I don't know**	**Total**
**NHS**	57 (24.3)	48 (20.4)	57 (24.3)	13 (5.5)	60 (25.5)	235
**Independent sector**	38 (44.7)	13 (15.3)	27 (31.8)	4 (4.7)	3 (3.5)	85

Table S4. If there is a formal agreement or policy in place with the other sector hospital on how you collaborate on clinical governance processes

Do you have a formal agreement or policy in place with the independent hospital on how you collaborate on clinical governance processes?	**Yes**	**No**	**I don't know**	**Total**
**NHS**	33 (15.9)	114 (54.8)	61 (29.3)	208
**Independent sector**	18 (22.2)	58 (71.6)	5 (6.2)	81

Table S5. Meeting with the other sector hospital on how you collaborate on clinical governance processes

Do you meet with the other sector hospital about clinical governance?	**Yes**	**No**	**I don't know**	**Total**
**NHS**	48 (23.1)	124 (59.6)	36 (17.3)	208
**Independent sector**	30 (37.5)	46 (57.5)	4 (5.0)	80

Table S6. How often they meet with staff responsible for clinical governance from the other sector hospital

On average, how often do you meet with staff responsible for clinical governance from the other sector hospital?	Once a week	Once a month	Once every 3 months	Once every 6 months	Once a year	Less than once a year	Never	I don't know	Total
**NHS**	1 (1.3)	12 (15.0)	17 (21.3)	5 (6.3)	6 (7.5)	4 (5.0)	8 (10.0)	27 (33.8)	80
**Independent sector**	2 (5.7)	9 (25.7)	14 (40.0)	3 (8.6)	2 (5.7)	1 (2.9)	1 (2.9)	3 (8.6)	35

Table S7. Types of meetings between the sectors about clinical governance

Are these meetings formal (e.g., minuted, contractually required) or informal (e.g., ad hoc)?	Formal	Informal	Both formal and informal	I don't know	Total
**NHS**	50 (27.9)	48 (26.8)	48 (26.8)	33 (18.4)	179
**Independent sector**	73 (96.1)	2 (2.6)	1 (1.3)	0 (0)	76

Table S8. Ratings of the clinical governance arrangements between the sectors

How would you describe the clinical governance arrangements you have with the other sector hospital?	Very good	Good	Acceptable	Poor	Very poor	I don't know	Total
**NHS**	9 (13.0)	17 (24.6)	17 (24.6)	2 (2.9)	1 (1.5)	23 (33.3)	69
**Independent sector**	7 (22.6)	15 (48.4)	6 (19.4)	1 (3.2)	0 (0)	2 (6.5)	31

Table S9. How often learning from patient safety incidents is shared with the other sector

Do you share learning from patient safety incidents with the other sector?	Always	Often	Sometimes	Rarely	Never	I don't know	Total

**NHS**	9 (13.0)	9 (13.0)	26 (37.7)	9 (13.0)	2 (2.9)	14 (20.3)	69
**Independent sector**	12 (37.5)	8 (25.0)	9 (28.1)	0 (0)	2 (6.3)	1 (3.1)	32

Table S10. Knowledge of which consultants are also working in the NHS trust/independent hospital

Do you know which of your consultants are also working at the other sector hospital?	**I know this for all of the consultants**	**I know this for most of the consultants**	**I know this for some of the consultants**	**I do not know this for any of the consultants**	**Total**
**NHS**	50 (27.9)	48 (26.8)	48 (26.8)	33 (18.4)	179
**Independent sector**	73 (96.1)	2 (2.6)	1 (1.3)	0 (0)	76

Table S11. How often the organisations get supporting information for scope of practice

Does your organisation get supporting information about the scope of practice for consultants who also work at the other sector hospital?	**Always**	**Often**	**Sometimes**	**Rarely**	**Never**	**I don't know**	**Total**
**NHS**	41 (23.2)	30 (17.0)	18 (10.2)	11 (6.2)	7 (4.0)	70 (39.6)	177
**Independent sector**	48 (63.2)	10 (13.2)	6 (7.9)	4 (5.3)	1 (1.3)	7 (9.2)	76

Table S12. Robustness of processes for verifying scope of practice

Our processes for verifying supporting information for scope of practice are robust	**Strongly agree**	**Agree**	**Neither agree nor disagree**	**Disagree**	**Strongly disagree**	**I don't know**	**Total**
**NHS**	7 (4.0)	33 (18.6)	25 (14.1)	11 (6.2)	64 (36.2)	37 (20.9)	177

**Independent sector**	21 (28.0)	31 (41.3)	3 (4.0)	0 (0)	6 (8.0)	14 (18.7)	75

Table S13. Level of confidence about the scope of practice of consultants who also work in the other sector

I am confident that our organisation knows about the scope of practice of consultants who also work at the other sector hospital	**Strongly agree**	**Agree**	**Neither agree nor disagree**	**Disagree**	**Strongly disagree**	**I don't know**	**Total**
**NHS**	9 (5.1)	45 (25.6)	28 (15.9)	10 (5.7)	41 (23.3)	43 (24.4)	176
**Independent sector**	23 (30.7)	35 (46.7)	2 (2.7)	0 (0)	3 (4.0)	12 (16.0)	75

Table S14. *How often revalidation decisions are delayed by not having supporting information about consultants' practice*

	**Always**	**Often**	**Sometimes**	**Rarely**	**Never**	**I don't know**	**Total**
**NHS** - Are revalidation decisions delayed by not having supporting information about consultants' practice in independent hospitals?	5 (2.8)	1 (0.6)	26 (14.7)	50 (28.3)	15 (8.5)	80 (45.2)	177
**Independent sector** - How often do consultants share information about their appraisals done by the NHS hospital?	58 (78.4)	8 (10.8)	1 (1.4)	0 (0)	3 (4.1)	4 (5.4)	74

Table S15. If the organisation has a policy on handling consultant concerns

Does your organisation have a policy on handling consultant concerns?	**Yes**	**No**	**I don't know**	**Total**
**NHS**	137 (78.7)	6 (3.5)	31 (17.8)	174
**Independent sector**	70 (93.3)	5 (6.7)	0 (0)	75

Table S16. If the policy includes a process for sharing information with the other sector hospital

Does the policy include a process for sharing information about concerns with the independent/NHS hospital?	**Yes**	**No**	**I don't know**	**Total**
**NHS**	87 (63.5)	24 (17.5)	26 (19.0)	137
**Independent sector**	65 (92.9)	3 (4.3)	2 (2.9)	70

Table S17. Frequency of sharing information from the respondent's organisation to the other sector hospital regarding consultant restrictions, exclusion and referral

		**Always**	**Often**	**Sometimes**	**Rarely**	**Never**	**I don't know**	**Total**
If there is a **significant concern** about one of your consultants, do you share information with the other sector hospital?	NHS	83 (47.7)	22 (12.6)	19 (10.9)	0 (0)	1 (0.6)	49 (28.2)	174
	Independent sector	67 (89.3)	3 (4.0)	2 (2.7)	0 (0)	0 (0)	3 (4.0)	75
If **interim restrictions** (temporary restrictions pending the outcome of an investigation) are placed on the practice of one of your consultants, do you share information with the other sector hospital?	NHS	96 (59.3)	12 (7.4)	8 (4.9)	1 (0.6)	1 (0.6)	44 (27.2)	162
	Independent Sector	64 (85.3)	2 (2.7)	3 (4.0)	0 (0)	0 (0)	6 (8.0)	75
If **substantial restrictions** (restrictions based on	NHS	98 (60.9)	12 (7.5)	4 (2.5)	0 (0)	1 (0.6)	46 (28.6)	161

conclusions from an investigation) are placed on the practice of one of your consultants, do you share information with the other sector hospital?	Independent Sector	65 (85.5)	4 (5.3)	2 (2.6)	0 (0)	0 (0)	5 (6.6)	76
If a consultant in your organisation is **suspended/excluded** for any reason, do you share information with the other sector hospital?	NHS	102 (63.4)	9 (5.6)	5 (3.1)	0 (0)	0 (0)	45 (28.0)	161
	Independent Sector	69 (90.8)	3 (4.0)	1 (1.3)	0 (0)	0 (0)	3 (4.0)	76
If a consultant in your organisation is **referred to the General Medical Council (GMC)**, do you share information with the other sector hospital?	NHS	72 (44.7)	19 (11.8)	20 (12.4)	2 (1.2)	1 (0.6)	47 (29.2)	161
	Independent Sector	64 (84.2)	3 (4.0)	1 (1.3)	2 (2.6)	0 (0)	6 (8.0)	76

Table S18. Frequency of sharing information from the other sector hospital to the respondent's organisation regarding consultant concerns, restrictions, exclusion and referral

		Always	Often	Sometimes	Rarely	Never	I don't know	Total
If there is a **significant concern** about one of your consultants at the other sector hospital, do they share this information with you?	NHS	25 (15.3)	29 (17.8)	21 (12.9)	5 (3.1)	5 (3.1)	78 (47.9)	163
	Independent sector	11 (14.5)	26 (34.2)	13 (17.1)	10 (13.2)	1 (1.3)	15 (19.7)	76
If the other sector hospital places **interim restrictions** on the practice of one of your consultants, do they share this information with you?	NHS	40 (24.7)	15 (9.3)	11 (6.8)	2 (1.2)	3 (1.9)	91 (56.2)	162
	Independent Sector	21 (27.6)	15 (19.7)	17 (22.4)	2 (2.6)	2 (2.6)	19 (22.4)	76
If the other sector hospital places **substantial restrictions** on the practice of one of your consultants, do they share this information with you?	NHS	41 (25.3)	16 (9.9)	10 (6.2)	1 (0.6)	3 (1.9)	91 (56.2)	162
	Independent Sector	24 (31.6)	16 (21.1)	16 (21.1)	2 (2.6)	2 (2.6)	16 (21.1)	76
If a consultant at the other sector hospital is **suspended/excluded** for any reason, do they share this information with you?	NHS	48 (29.8)	12 (7.5)	8 (5.0)	1 (0.6)	3 (1.9)	89 (55.3)	161
	Independent Sector	30 (39.5)	17 (22.4)	10 (13.2)	1 (1.3)	1 (1.3)	17 (22.4)	76
If the other sector hospital **refers one of your**	NHS	28 (17.5)	17 (10.6)	9 (5.6)	1 (0.6)	2 (1.3)	103 (64.4)	160

**consultants to the GMC**, do they share this information with you?	Independent Sector	28 (36.8)	15 (19.7)	9 (11.8)	2 (2.6)	2 (2.6)	20 (26.3)	76

Table S19. GMC sharing information about referrals, restrictions and suspensions

		Always	Often	Sometimes	Rarely	Never	I don't know	Total
If a GMC referral is made by the other sector hospital, does the GMC inform your hospital directly?	NHS	55 (34.2)	17 (10.6)	10 (6.2)	2 (1.2)	0 (0)	77 (47.8)	161
	Independent Sector	35 (46.1)	10 (13.2)	5 (6.6)	0 (0)	1 (1.3)	25 (32.9)	76
If the GMC restricts the practice or suspends the licence of a consultant from the other sector hospital, does the GMC inform you directly?	NHS	69 (43.1)	10 (6.3)	13 (8.1)	0 (0)	1 (0.6)	67 (41.9)	160
	Independent Sector	39 (51.3)	6 (7.9)	5 (6.6)	0 (0)	1 (1.3)	25 (32.9)	76

Table S20. Who information about concerns, restrictions, exclusion and referral at the other sector hospital

Who do you share information with at the other sector hospital?	NHS	Independent sector
Chief Executive	29 (12.3)	7 (8.2)
Chief Medical Officer	65 (27.7)	34 (40.0)
Chief Nurse	10 (4.3)	3 (3.5)
Clinical Governance Lead	18 (7.7)	9 (10.6)
Director of Clinical Services	15 (6.4)	5 (5.9)
Director of Nursing	4 (1.7)	0 (0)
Hospital Director	27 (11.5)	2 (2.4)
Matron	1 (0.4)	0 (0)
Medical Director	34 (14.5)	25 (29.4)
Responsible Officer	52 (22.1)	43 (50.6)
Other	18 (7.7)	6 (7.1)

Table S21. How information about concerns, restrictions, exclusion and referral is shared with the other sector hospital

What is the main method you use to share information with the other sector hospital?	NHS trusts	Independent sector
Medical Practice Information Transfer (MPIT)	32 (21.1)	15 (19.7)

Telephone	17 (11.2)	11 (14.5)
Email	53 (34.9)	31 (40.8)
Other	17 (11.2)	10 (13.2)
I don't know	33 (21.7)	9 (11.8)
**Total**	**152**	**76**

Table S22. Fulfilment of consultant professional obligation to inform other organisations about significant concerns, restrictions, exclusions/suspensions

Do consultants fulfil their professional obligation to inform other organisations about significant concerns, restrictions, exclusions/suspensions (including the other sector hospital)?	**Always**	**Often**	**Sometimes**	**Rarely**	**Never**	**I don't know**	**Total**
**NHS**	20 (13.1)	46 (30.1)	17 (11.1)	1 (0.7)	0 (0)	69 (45.1)	153
**Independent sector**	16 (21.1)	28 (36.8)	19 (25.0)	0 (0)	0 (0)	13 (17.1)	76

Table S23. Knowledge about the capacity and capability of the independent sector to treat deteriorating patients that require an escalation in care

	**Very good**	**Good**	**Acceptable**	**Poor**	**Very poor**	**I don't know**	**Total**
**NHS** - My knowledge about the capacity and capability of the independent hospital to treat deteriorating patients that require an escalation in care is...	15 (10.4)	29 (20.1)	41 (28.5)	9 (6.3)	12 (8.3)	38 (26.4)	144
**Independent sector** - The NHS hospitals knowledge about the capacity and capability of the independent hospital to treat deteriorating	12 (26.1)	18 (39.1)	9 (19.6)	6 (13.0)	0 (0)	1 (2.2)	46

patients that require an escalation in care is…	

Table S24. What the NHS hospital knows about the capacity and capability of the independent hospital and what the independent hospital thinks the NHS hospital knows about the capacity and capability of their hospital

	NHS - Which of the following do you know about the independent hospital?	Independent sector - Which of the following do you think the NHS hospital knows about the capacity and capability of your hospital?
How many inpatient beds they/you have	54 (23.0)	19 (22.4)
How many operating theatres they/you have	53 (22.6)	20 (23.5)
Whether they/you have their own critical care facilities	76 (32.3)	32 (37.7)
What out of hours medical cover they/you have	53 (22.6)	25 (29.4)
None of these	64 (27.2)	9 (10.6)

Table S25. *If the organisation has a policy on sharing or transferring patient clinical information and records*

Is there any form of written policy or agreement in place with the independent/NHS hospital on how you share or transfer patient clinical information and records?	**Yes**	**No**	**I don't know**	**Total**
**NHS**	32 (23.4)	38 (27.7)	67 (48.9)	137
**Independent sector**	52 (70.3)	16 (21.6)	6 (8.1)	74

Table S26. Sending and receiving timely information on the patient when there is an urgent transfer

	**Always**	**Often**	**Sometimes**	**Rarely**	**Never**	**I don't know**	**Total**
**NHS** - When there is an urgent transfer of a patient from the independent hospital to your organisation, do you receive timely and adequate information on the patient from the independent hospital?	20 (14.7)	47 (34.6)	15 (11.0)	3 (2.2)	1 (0.7)	50 (36.8)	136
**Independent sector** - When there is an urgent transfer of a patient from your organisation to the NHS hospital, do you send timely and adequate information about the patient to the NHS hospital?	70 (94.6)	3 (4.1)	0 (0)	0 (0)	0 (0)	1 (1.4)	74

Table S27. Providing and receiving information about subsequent treatment and outcomes following an urgent transfer

	Always	Often	Sometimes	Rarely	Never	I don't know	Total
**NHS** - When there is an urgent transfer of a patient from the independent hospital to your organisation, do you provide information to the independent hospital about subsequent treatment and outcomes?	15 (11.0)	36 (26.5)	19 (14.0)	3 (2.2)	1 (0.7)	62 (45.6)	136
**Independent sector** - When there is an urgent transfer of a patient from your organisation to the NHS hospital, are you able to find out about their subsequent treatment and outcomes?	30 (41.1)	22 (30.1)	16 (21.9)	5 (6.9)	0 (0)	0 (0)	73

Table S28. Impact of the COVID19 pandemic on clinical governance with the other sector hospital

During the COVID19 pandemic when independent hospitals were providing more NHS funded elective care, did the arrangements for clinical governance between your organisation and the other sector hospital change?	Yes	No	I don't know	Total
**NHS**	38 (27.1)	42 (30.0)	60 (42.9)	140
**Independent sector**	25 (33.8)	28 (37.8)	21 (28.4)	74

Table S29. Whether changes as a result of COVID19 are still in place

Are these changes still in place?	**Yes**	**No**	**I don't know**	**Total**
**NHS**	5 (13.2)	22 (57.9)	11 (29.0)	38
**Independent sector**	10 (40.0)	15 (60.0)	0 (0)	25
